# Mechanically durable liquid-impregnated honeycomb surfaces

**DOI:** 10.1038/s41598-017-06621-1

**Published:** 2017-07-20

**Authors:** Philip S. Brown, Bharat Bhushan

**Affiliations:** Nanoprobe Laboratory for Bio- & Nanotechnology and Biomimetics (NLBB), The Ohio State University, 201W. 19th Avenue, Columbus, OH 43210-1142 USA

## Abstract

Liquid repellent surfaces typically work by keeping the fouling liquid in a metastable state, with trapped pockets of air between the substrate and the liquid. An alternative method with greater long-term stability utilizes liquid-impregnated surfaces, where the liquid being repelled slides over an immiscible liquid immobilized on a porous surface. Here, we report a method for creating honeycomb surfaces amenable to liquid-impregnation. Polystyrene dissolved in a water-immiscible, volatile solvent was deposited in a humid environment on a variety of substrates to achieve the necessary porosity. Evaporative cooling results in condensation of water in a breath figure array of droplets, forming a sacrificial template for the drying polymer film. These honeycomb surfaces were further functionalized with fluorosilane and dipped in the lubricating liquid to result in a durable, liquid-repellent surface. These surfaces were found to exhibit repellency towards water and oils with extremely low tilt angles due to the smooth liquid–liquid contact between the lubricating liquid and the liquid being repelled.

## Introduction

A range of desirable surface properties such as anti-fouling, self-cleaning, and anti-smudge repellency all rely on altering both the chemistry and roughness of a surface to achieve liquid repellency^1, 2^. Extreme water repellency, also known as superhydrophobicity, is where the contact angle of water on a surface is greater than 150° and the contact angle hysteresis (difference between advancing and receding contact angles) is less than 2°. This allows the liquid droplet to roll off the surface with no contamination. This repellency is typically achieved by roughening a hydrophobic surface, increasing the solid surface area in contact with the droplet^3^. Alternatively, air can become trapped between the surface and the liquid^4^.

Liquid repellency becomes more difficult when the surface tension of the liquid to be repelled is low. This is true for oils, since oil droplets typically exhibit contact angles of <90° on flat surfaces (oleophilic). However, high repellency via the Cassie-Baxter state of wetting can still be achieved through the use of re-entrant surface features, where the asperities create an overhang (i.e. become narrower closer to the surface)^5–9^. However, such a configuration whereby air is trapped between the droplet and surface is only metastable state and, via applied pressure or surface vibration, the liquid will eventually penetrate into the roughness and fully wet the surface.

An alternate method of creating liquid-repellent surfaces is to take inspiration from the *Nepenthes* pitcher plants, which features a microstructured surface that is wet by netar and rainwater to result in a continuous liquid film^10^. When wet, this region becomes extremely slippery and insects aquaplane across the surface and fall into the pitcher. There are several examples of pitcher plant-inspired, liquid-impregnated surfaces. These previous examples have several drawbacks that potentially limit their applicability to a range of scenarios. Teflon nanofibres and epoxy-molded nanoposts have previously been utilized as the required porous solid surface^11^. However, such examples are unsuitable for certain real world applications due to their composition, fragility, and cost of fabrication.

Other previous examples rely on specific substrates. One such embodiment requires an oxide substrate for colloidal templating of a highly ordered porous monolayer^12^. Other liquid-impregnated repellent surfaces have been reported on nano-textured alumina^13^ and electrodeposited polypyrrole nanostructures^14^. One substrate-independent method potentially more suited to a range of applications utilizes a spray coated porous wax layer^15^. However, the durability of a porous wax coating remains unclear. More recently, a mechanically durable liquid-repellent polypropylene coating was achieved through the creation of a liquid-impregnated porous polymer surface. Porous polypropylene was created through the use of a solvent–nonsolvent polymer solution^16^.

One alternative approach for the fabrication of porous surfaces is to utilise breath figures^17, 18^. Breath figures are two-dimensional hexagonally packed arrays of water droplets condensed onto a cooled surface. Such a breath figure can form on a drying polymer film surface, so long as the solvent used to cast the film is immiscible with water. Evaporation of the solvent leads to evaporative cooling of the film surface, resulting in water condensation and the formation of a breath figure array. Droplet coalescence is limited either by the increasing viscosity of the drying polymer film or by precipitation of the polymer at the water–solvent interface^19^. This array of water droplets acts as a template for the drying polymer solution leading to the formation of the porous surface structure once the solvent and water have fully evaporated. Based on their appearance, such breath figure-templated surfaces are commonly called honeycomb surfaces.

Polystyrene is a common polymer used in the creation of honeycomb surfaces^20–22^. Here, polystyrene honeycomb films are created on glass and polymer substrates and further treated with UV irradiation and fluorosilane coupling to ensure the lubricating liquid will remain impregnated within the polymer structure and no preferential dewetting will occur when the liquid to be repelled is added to the surface. The chemically modified honeycomb surfaces were then dipped into the lubricating liquid. The repellency of these liquid-impregnated honeycomb surfaces was tested against water and hexadecane. The mechanical durability of these surfaces was investigated through the use of macrowear experiments. Previous work has demonstrated the ability of liquid-impregnated honeycomb surfaces for the repellency of various liquids^23^. However, no durability study was conducted. These liquid-repellent honeycomb surfaces will be of interest for a range of applications such as in packaging, where it is desirable for the product to be completely removed with little to no fouling of the walls of the container, reducing wastage and improving the recyclability of the container.

## Methods

**Figure 1:**
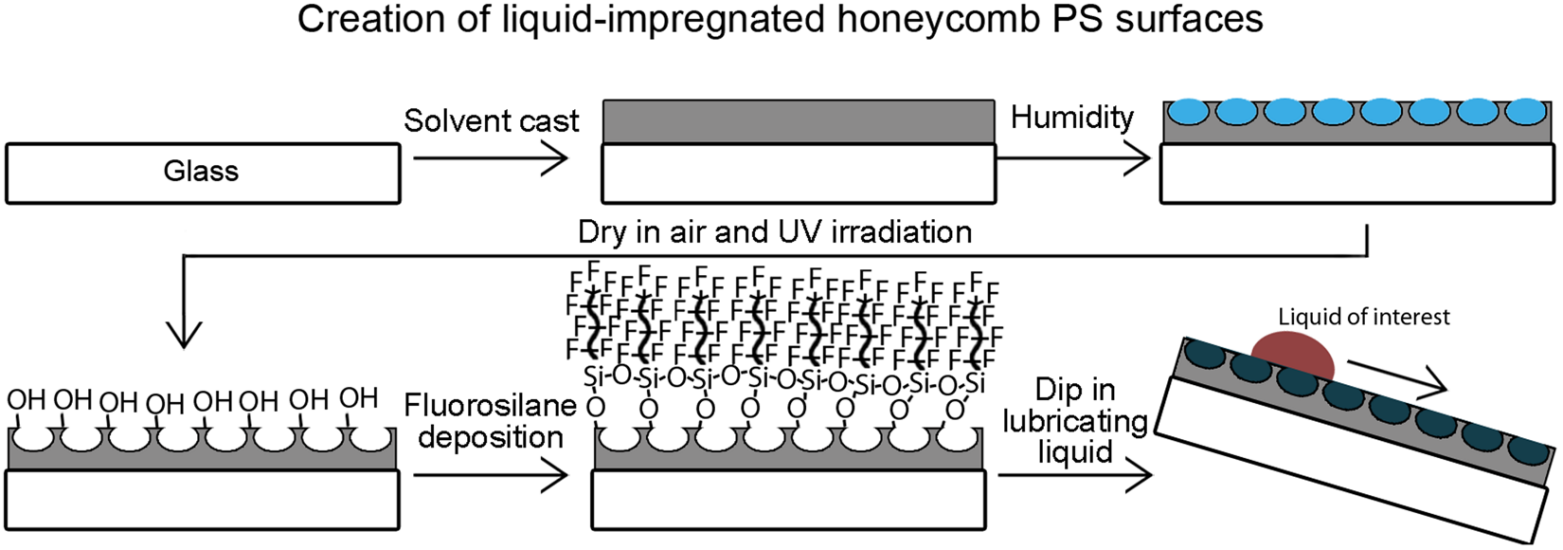
Schematic to show the creation of liquid-impregnated honeycomb surfaces. Polymer dissolved in a water-immiscible, volatile solvent is first cast onto a substrate in a humid environment. Evaporative cooling leads to condensation of water and the formation of a breath figure array of droplets. The droplet array acts as a sacrificial template for the drying polymer film.

In order to achieve the porous structures required for creating liquid-impregnated surfaces, the surfaces described in this paper comprise polystyrene coatings cast in a volatile, water-immiscible solvent and allowed to dry in a humid environment, Fig. 1. The honeycomb surface is then activated using UV irradiation and treated with fluorosilane to better to ensure the impregnating liquid will preferably wet the surface. The surface is then dipped in the impregnating liquid to result in a liquid repellent surface.

### Samples

Glass slides (Fisher Scientific) and polypropylene sheet (PP, ASTM D4101–0112, SPI) cut to dimensions of 15 by 15 mm were used as substrates. 0.2 g of polystyrene (Mw ~ 350,000, Sigma Aldrich) was dissolved in 10 mL chloroform (Mallinckrodt) at room temperature. Once the polymer was fully dissolved, a droplet of the solution was added to the glass surface at room temperature and ambient humidity (54% RH) and the surface was dried in air. To activate the polymer surface for silane attachment, samples were UV irradiated for 30 min (15 W, λ_max_ = 254 nm). Samples were fluorinated via chemical vapor deposition of a silane (to lower surface energy), which was required in order to ensure preferential wetting by the lubricating liquid. One drop of trichloro(1H, 1H, 2H, 2H-perfluorooctyl) silane (fluorosilane, Sigma Aldrich) was deposited next to the samples which were covered and left for 2 h. The sample was then dipped into the impregnating liquid, in this instance a perfluoropolyether (Krytox GPL 102, Dupont) with a chemical structure of F-(CF(CF_3_)-CF_2_-O)_n_-CF_2_CF_3_ where n = 10–60, a surface tension of 16–20 mN m^−1^, and a viscosity of 38 cSt.

### Contact angle and tilt angle

For contact angle data, droplets of both water (5 µL, surface tension 72 mN m^−1^) and n-hexadecane (5 µL, 99%, Alfa Aesar, surface tension 27 mN m^−1^ (ref. 24) were dispensed onto the surface of samples using a goniometer (Model 290, Ramé-Hart Inc.) with the resulting droplet shape analyzed with DROPimage software. Tilt angles were determined by moving the surface until the 5-µL droplet was observed to slide off. All angles reported are the average of five separate measurements performed on different areas of a sample.

### Optical imaging

Optical images were taken with a CCD camera (Nikon Optihot-2) to determine the topography of the polystyrene samples.

### Wear experiments

The mechanical durability of the surfaces was examined through macrowear experiments performed with an established procedure of using a ball-on-flat tribometer, initially described elsewhere^2, 8, 25^. Briefly, a 3-mm sapphire ball with an applied load of 10 mN normal to the surface was put into reciprocating motion for 200 cycles (stroke length = 6 mm, average linear speed = 1 mm s^−1^). Optical images were taken before and after the experiment to track the formation of a wear scar.

Contact pressures were calculated based on Hertz analysis^25^. For the surface, an elastic modulus of 3 GPa and a Poisson’s ratio of 0.35 were used^26^. For the sapphire ball, an elastic modulus of 390 GPa and Poisson’s ratio of 0.23. The mean contact pressure was calculated as 14 MPa.

## Results and Discussion

**Figure 2:**
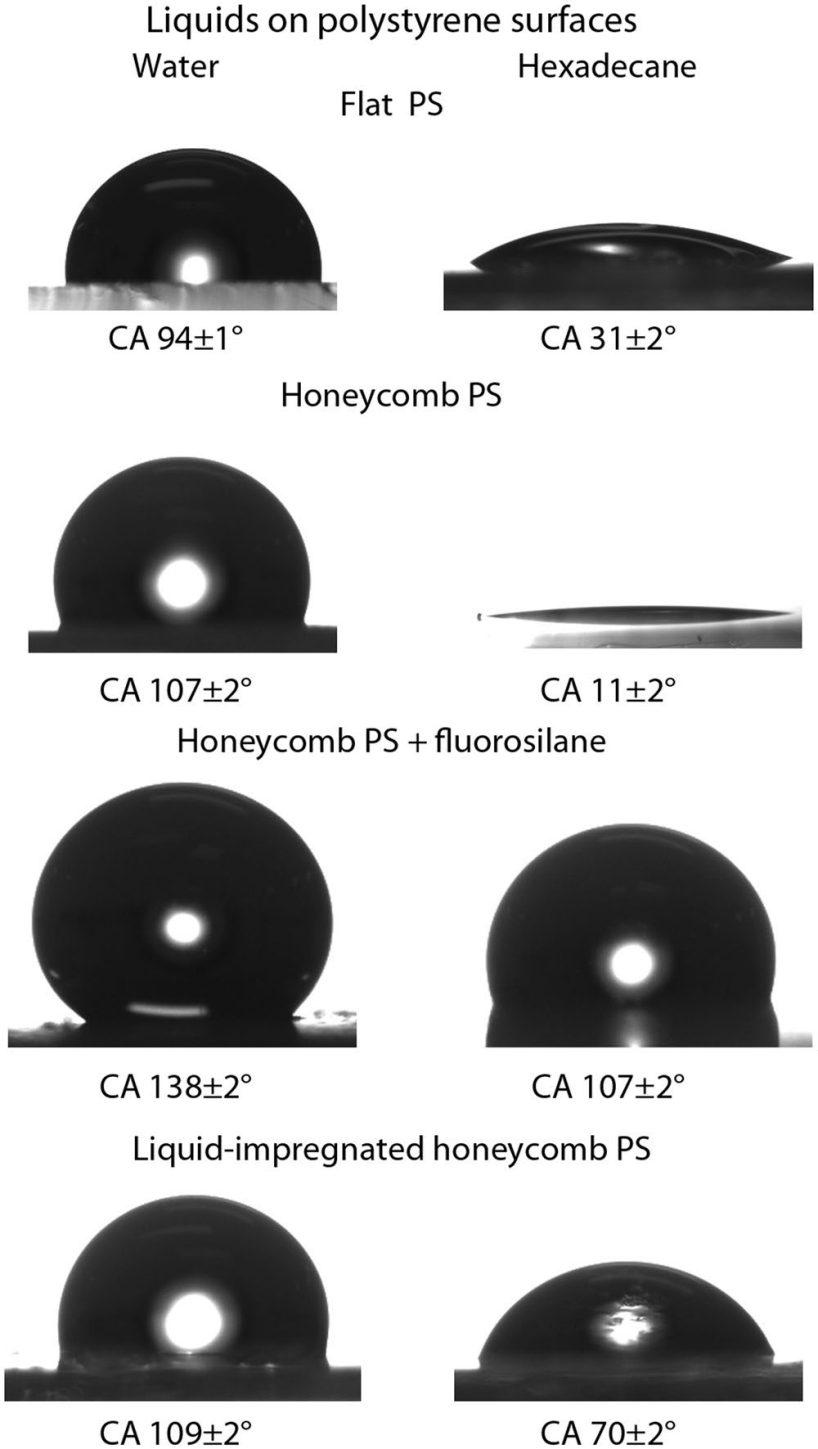
Contact angle images for droplets water and hexadecane on polystyrene surfaces.

**Figure 3:**
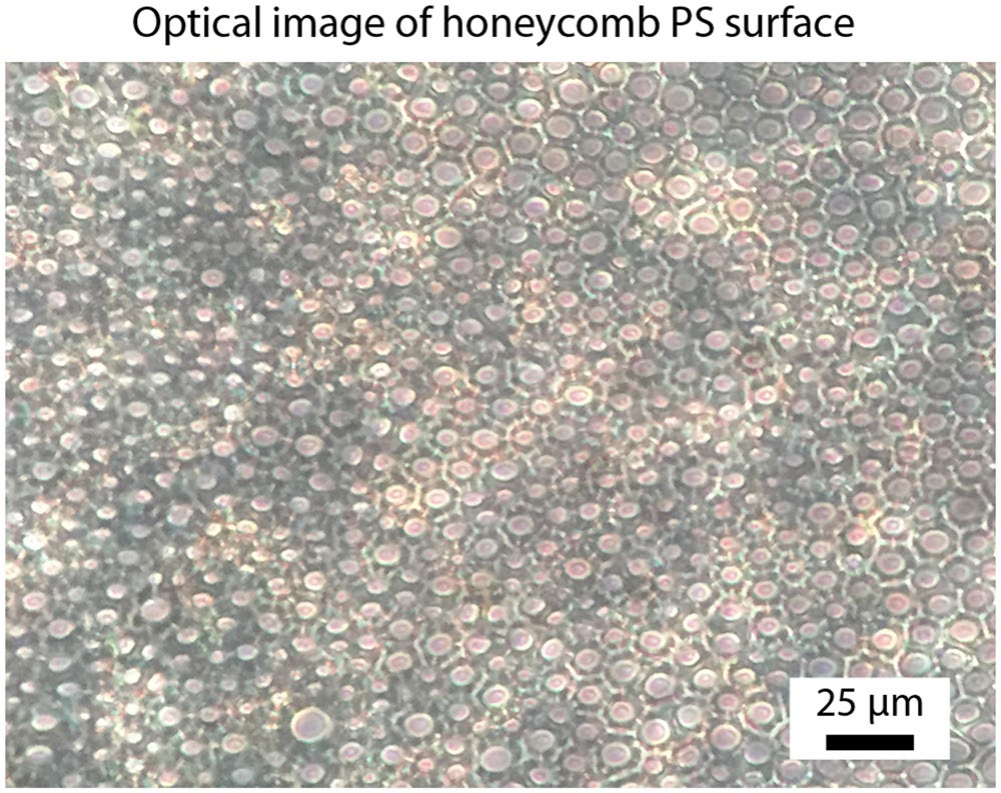
Optical images of a honeycomb surface on glass after solvent casting polystyrene from a water-immiscible, volatile solvent in a humid environment.

Flat polystyrene (PS) is found to be slightly hydrophobic with water contact angles of 94 ± 1°, Table 1 and Fig. 2. In order to create the porous polymer surface, polystyrene was dissolved in a water immiscible, volatile solvent. A drop of the solution was cast onto a substrate and dried in a humid environment at room temperature. Evaporative cooling of the drying polymer film results in the condensation of water droplets and the formation of a breath figure. This array of water droplets acts as a sacrificial template for the drying polymer film and, once evaporation of the solvent and water is complete, results in a porous, honeycomb surface structure, Fig. 3. Fully dried, the polystyrene honeycomb surface was found to have a water contact angle of 107 ± 2° due to the increase in surface roughness.

**Table 1:** Comparison of static contact angles and tilt angles for water and hexadecane droplets deposited on polystyrene surfaces.

Surface	Water		Hexadecane	
	Contact angle (°)	Tilt angle (°)	Contact angle (°)	Tilt angle (°)
Flat PS	94 ± 1	N/A	31 ± 2	N/A
Honeycomb PS	107 ± 2	N/A	11 ± 2	N/A
Honeycomb PS + fluorosilane	138 ± 2	N/A	107 ± 2	N/A
Honeycomb PS + fluorosilane + lubricating liquid	109 ± 2	2 ± 1	70 ± 1	4 ± 2

**Figure 4:**
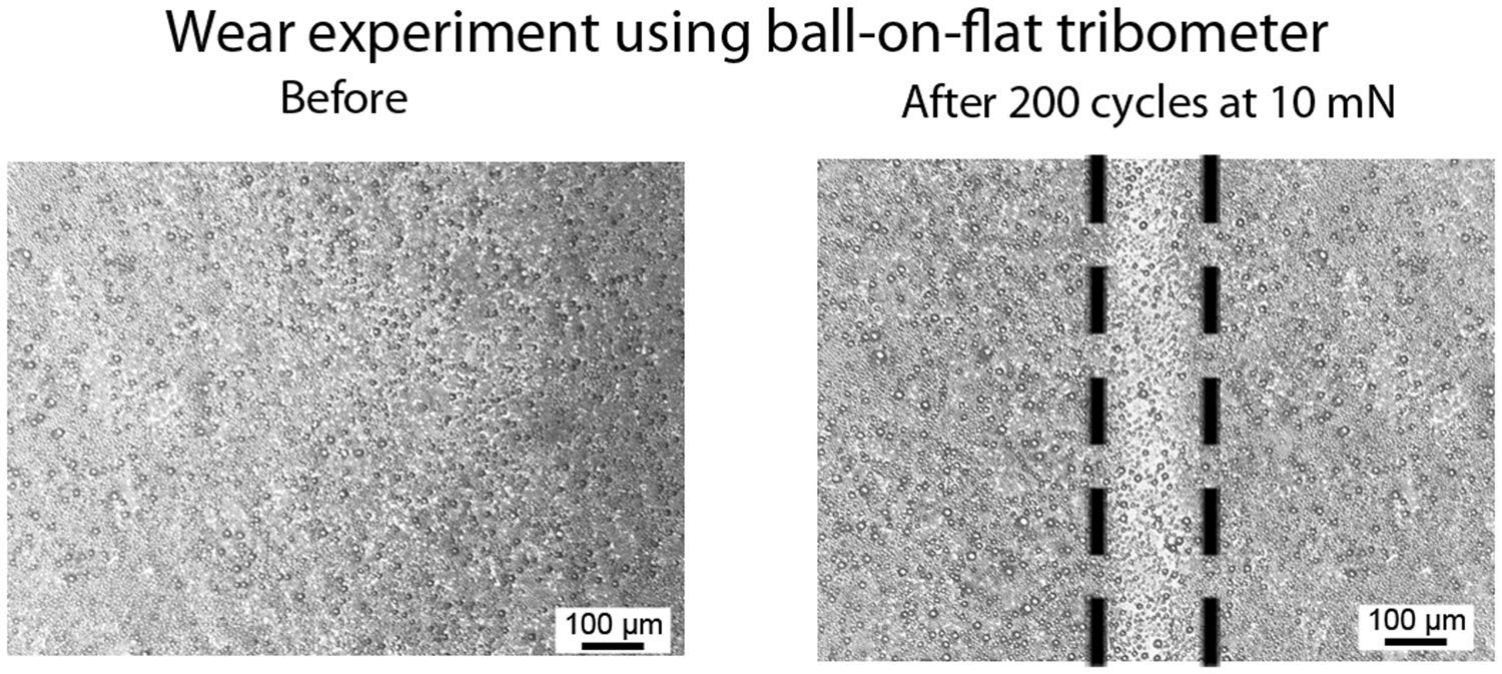
Optical micrographs before and after wear experiments using ball-on-flat tribometer using a 3-mm diameter sapphire ball at 10 mN loading on a honeycomb surface.

The mechanical durability of the polystyrene honeycomb surface was investigated through the use of tribometer wear experiments and the resulting optical images, showing a portion of the wear track, are displayed in Fig. 4. The wear experiments were carried out with a load of 10 mN, with the tribometer put in reciprocating motion for 200 cycles. The images confirm that the polymer coating is not removed from the glass substrate. The density of the honeycomb structure appears to decrease in the wear location due to plastic deformation of the polymer. However, the porous structure is not completely destroyed, allowing for the impregnating liquid to remain in the wear region. It is believed that these surfaces can likely be more durable than many other examples of liquid-impregnated surfaces, which typically rely on poorly adhered wax coatings^15^ or delicate surface structures^11^.

For the lubricating liquid to fully penetrate the porous surface, the chemistry of the honeycombs was altered, ensuring favorable wetting and no preferential dewetting when another liquid is added on top of the lubricating liquid layer. The polystyrene honeycomb coating was activated via UV irradiation to activate the surface for silane attachment. Following fluorosilane treatment, the polystyrene honeycomb surface displayed water contact angles of 138 ± 2° and hexadecane contact angles of 107 ± 2°, Fig. 2. This altering of the surface energy is necessary to ensure that the lubricating liquid, in this case a lower surface tension fluorinated oil, will remain impregnated in the honeycomb structure and will not be preferentially replaced by the liquid to be repelled.

Finally, the honeycomb surface was dipped into the lubricating liquid. Following this, the liquid-impregnated surface exhibited water contact angles of 109 ± 2° and hexadecane contact angles of 70 ± 2°, Fig. 2. However, due to the presence of the lubricating liquid, the surface displays very low tilt angles of 2 ± 1° and 4 ± 2° for water and hexadecane respectively, Table 1. Because the low tilt angles are a product of the homogeneity of the liquid–liquid interface, the surface tension of the liquid being repelled has little effect on the repellency of the surface.

**Figure 5:**
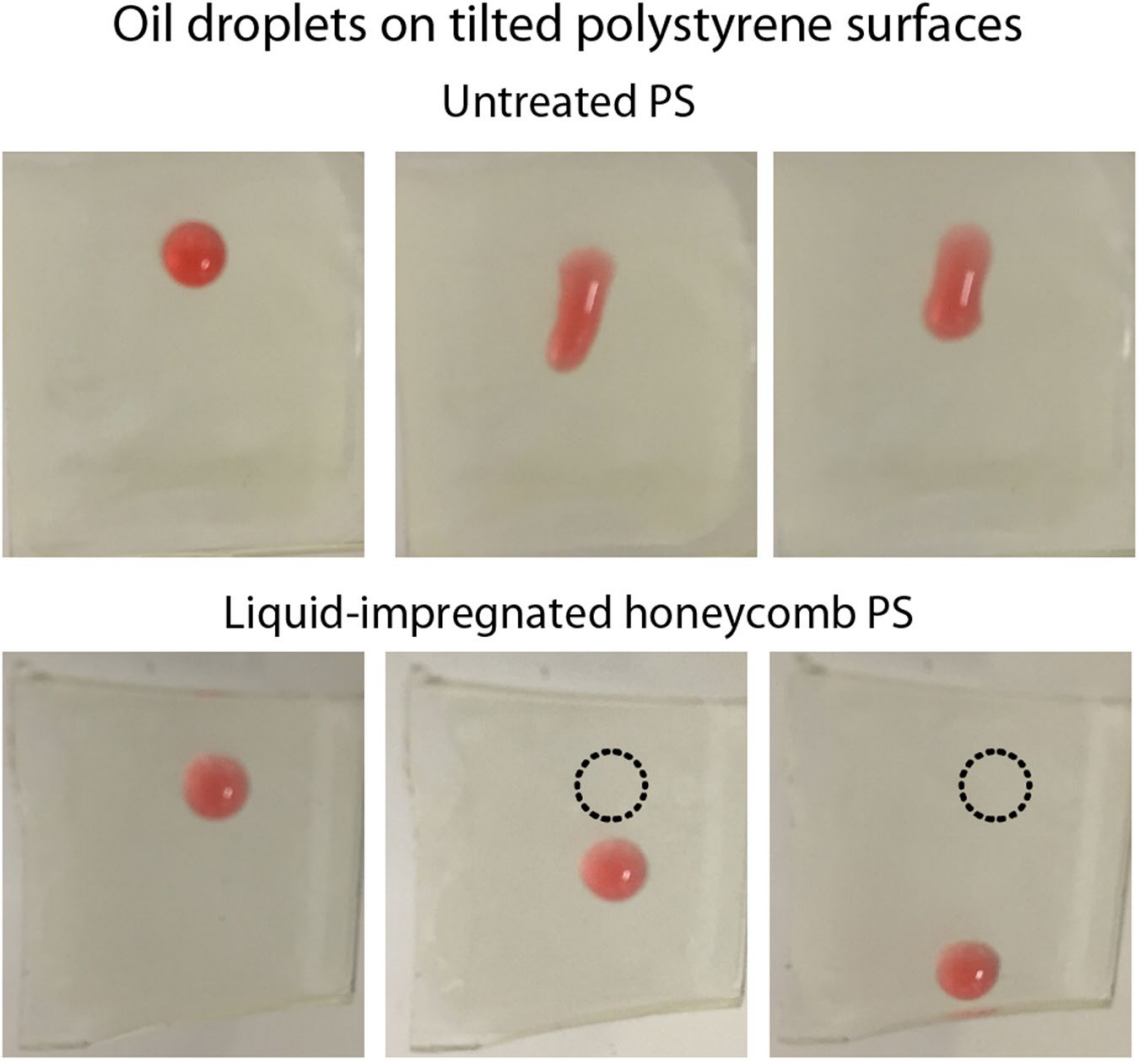
Photographs demonstrating hexadecane repellency of liquid-impregnated honeycomb surface compared to a fluorinated honeycomb sample.

The low tilt angle means that liquid droplets placed on the surface are able to slide over the surface easily. In Fig. 5, droplets of hexadecane were added to fluorosilane-treated honeycomb and liquid-impregnated honeycomb surfaces. Hexadecane droplets on the fluorosilane-treated surface are not easily removed when the surface is titled due to high hysteresis and droplet pinning. In contrast, as the liquid-impregnated surface is tilted, the hexadecane droplet slides across the surface with very little resistance. The red dye present in the hexadecane droplet helps to confirm that the vacated area of the surface is not contaminated by the hexadecane. Further wear experiments carried out on honeycomb surfaces containing the lubricating liquid did not result in any change in the repellent properties of the surface, with droplets of hexadecane sliding over the wear location with no noticeable degradation in the repellency.

In certain applications, liquid-impregnated surfaces can exhibit greater long-term repellency than traditional liquid-repellent surfaces, the repellency of which is dependent on metastable states and trapped air. For instance, liquid-impregnated surface treatments could be better suited for applications where the contaminant liquid is in constant contact with the surface for extended periods of time or where the substrate is subject to vibration.

## Conclusions

Liquid-repellent, slippery surfaces have been created on glass and polymer substrates via the formation of a honeycomb structure. Following UV activation and fluorosilane coupling to reduce the surface energy of the honeycombs, the substrate was dipped in a lubricating liquid, which became impregnated within the pores. This lubricating liquid layers repels other liquids placed on the surface through immiscible liquid–liquid contact. This results in very low tilt angles with droplets of both water and hexadecane sliding across the surface with no contamination. Such liquid-repellent surfaces will be more stable than repellent surfaces relying on the Cassie-Baxter state of wetting, where the liquid droplet being repelled is in a metastable state.
